# Multi-Biomarkers Panel in Identifying Benign and Malignant Lung Diseases and Pathological Types of Lung Cancer

**DOI:** 10.7150/jca.85846

**Published:** 2023-06-26

**Authors:** Lige Yao, Yanli Li, Qin Wang, Tian Chen, Jiayin Li, Yingjie Wang, Liuyan Zhang, Li Su, Lanqing Li, Qinqin Lou, Fang Li, Jiali Zhao, Junli Gao, Junshun Gao, Huiqin Li

**Affiliations:** 1 The Third People's Hospital of Zhengzhou, Zhengzhou, China.; 2 The First People's Hospital of Shangqiu, Shangqiu, China.; 3 Key Laboratory of Precision Medicine in Diagnosis and Monitoring Research of Zhejiang Province, Hangzhou, China.; Lige Yao and Yanli Li contributed equally to the study.

**Keywords:** lung cancer, benign and malignant lung diseases, cancer diagnosis, multi-biomarkers detection, serum protein markers

## Abstract

With the discovery of many tumor markers, there are new strategies for the early diagnosis and treatment of lung cancer and the prediction of prognosis. We examined the multi-protein markers panel (4MP, consisting of Pro-SFTPB, CA125, Cyfra21-1, and CEA) diagnosis performance in differentiating benign and malignant lung diseases and identifying pathological types of lung cancer. Meantime, the complementary performance of three conventional tumor markers (NSE, SCC, and Pro-GRP) for 4MP was assessed. A total of 294 patients with lung cancer or benign lung disease are contained in this study. The AUCs of 4MP and 7MP (NSE, SCC, Pro-GRP, and 4MP) in distinguishing benign lung disease and lung cancer were 0.808 and 0.832, respectively. In distinguishing SQCLC and SCLC, the AUCs were 0.716 and 0.985, respectively. In distinguishing LADC and SCLC, the AUCs were 0.849 and 0.998, respectively. This study demonstrated that 4MP can distinguish lung cancer from benign disease. Traditional biomarkers NSE, SCC, and Pro-GRP can significantly improve the performance of 4MP in the differentiation of LADC, SQCLC, and SCLC, which is expected to contribute to the accurate diagnosis and personalized treatment of patients.

## Introduction

The respiratory tract is easily infected and causes a variety of diseases due to contact with the outside air at any time. Lung cancer is one of the most serious diseases of the respiratory tract 1. The 2022 American Cancer Report states that lung cancer has the highest mortality 2. In China, lung cancer is also the cancer with the highest incidence rate (17.9%) and mortality (23.8%) 3. Small-cell lung cancer (SCLC) and non-small-cell lung cancer (NSCLC) are two major subtypes of lung cancer 4. NSCLC accounts for about 85% of all lung cancer types, which can be divided into lung adenocarcinoma (LADC) and squamous cell lung cancer (SQCLC) 5. The majority of patients have already developed metastasis once diagnosed, which is the main reason for the poor prognosis of lung cancer 6. Thus, the early detection of lung cancer and timely clinical intervention is the key to reducing the mortality of primary lung cancer.

In recent years, with the discovery of many tumor markers, there are new strategies for the early diagnosis and treatment of lung cancer and the prediction of prognosis 7. However, TNM staging is still the most critical indicator to predict the survival time of lung cancer patients 8. A high misdiagnosis rate is one of the main reasons for late staging and poor prognosis of patients 9. Research shows that in some areas of China, the misdiagnosis rate of lung cancer among people under 40 years old can reach more than 50%, mainly misdiagnosed as pneumonia, followed by benign lung diseases such as tuberculosis 10. The early symptoms of lung cancer are cough, dyspepsia, and fever, similar to pneumonia. Due to the lack of specificity of chest X-ray examination, obstructive pneumonia, and atelectasis often mask the characteristics of lung tumor lesions. Low-dose spiral CT (LDCT), as the standard method for early diagnosis of lung cancer, still has limitations such as high false positive rate and radiation exposure 11, 12. Serological markers are important for the differential diagnosis of lung cancer and pneumonia and are ideal detection objects. Serological screening and lung cancer diagnosis mainly depend on tumor markers such as carcinoma embryonic antigen (CEA) and neuron-specific enolase (NSE) 13. In this regard, many multi-analysis teams composed of circulating proteins and tumor-related antibodies have been developed. Although they have high sensitivity and specificity in distinguishing lung cancer patients from healthy people, they are not satisfactory in the differential diagnosis of lung cancer and benign lung diseases. Therefore, the development of a panel that can effectively distinguish between lung cancer and benign lung disease is of great significance for early lung cancer screening and diagnosis 14.

**Figure 1:**
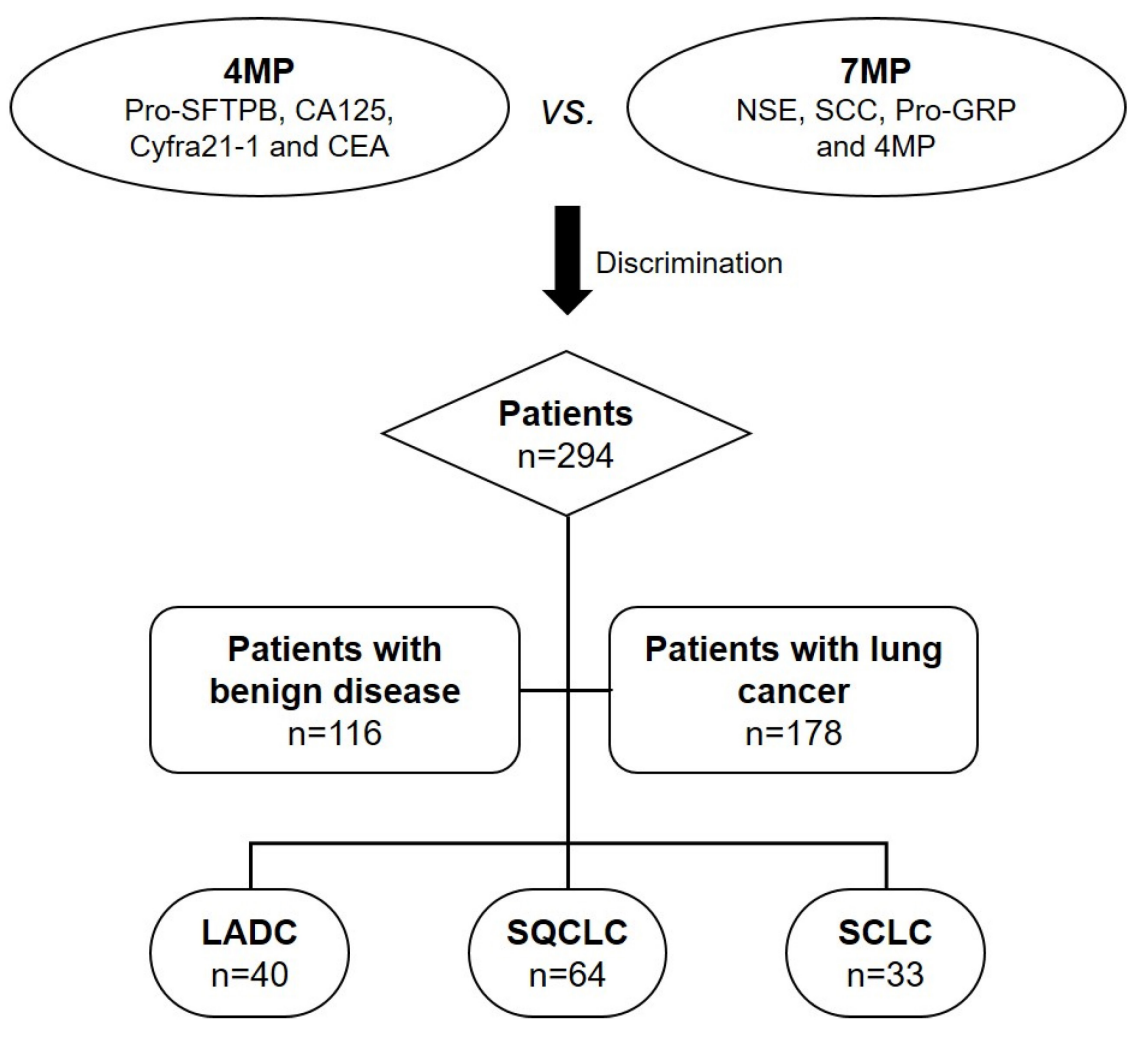
Flowchart of multi-protein markers panel in differentiating benign and malignant lung diseases and identifying pathological types of lung cancer. LADC: lung adenocarcinoma; SQCLC: squamous-cell carcinoma; SCLC: small cell lung cancer.

In this work, we proposed a multi-biomarker panel (4MP) combining pro-surfactant protein B (Pro-SFTPB), carbohydrate antigen 125 (CA125), cytokeratin 19 fragmentCyfra21-1, and CEA to distinguish lung cancer from benign lung disease in 294 patients. In particular, this panel performed better than individual biomarkers in determining early cancer from benign lung disease. Surprisingly, we found that the multi-biomarker panel (7MP) combining the conventional markers NSE, squamous cell carcinoma antigen (SCC), pro-gastrin-releasing peptide (Pro-GRP), and 4MP could significantly improve the performance of 4MP in differentiated LADC, SQCLC, and SCLC, which may contribute to achieving personalized precision treatment. **Figure 1** showed the flowchart of the multi-protein markers panel in differentiating benign and malignant lung diseases and identifying pathological types of lung cancer. We hope the results of this study lay the foundation for large-scale clinical trials before clinical transformation, and further provide clinical feasibility for early diagnosis of lung cancer in China.

## Materials and Methods

### Study subjects

Blood samples were collected from The Third People's Hospital of Zhengzhou and The First People's Hospital of Shangqiu. From January 2022 to December 2022, a total of 294 patients were recruited, including 116 patients with benign lung disease and 178 patients with lung cancer. The benign lung diseases contained mainly pneumonia, pulmonary fibrosis, pulmonary obstruction, and pulmonary abscess. All patients in the group should meet the following criteria: (a) no family history of lung cancer or other malignant tumors; (b) no radiotherapy or chemotherapy; and (c) no extrathoracic malignant diseases. At the same time, the clinical data of the patients, including age, sex, medical history, pathological diagnosis, and imaging findings, were collected and recorded in the database. This study is in line with the ethical guidelines of the Helsinki Declaration and has been approved by the Ethics Review Committee of the Third People's Hospital of Zhengzhou (2021-01-021-K01).

### Determination of serum biomarkers levels

The ADVIA Centaur^®^XP automatic immunofluorescence analyzer (Siemens Healthcare Diagnostics Inc, USA) was used to detect serum biomarkers levels, and their cut-off values refer to the specifications. The Pro-SFTPB detection was used an in-house developed ELISA kit with a mouse monoclonal antibody targeting the N-terminus of Pro-SFTPB. CEA and CA125 were detected using a multiplex assay kit from EMD Millipore. CYFRA21-1 detected by a single assay kit from R&D Systems (Minneapolis, MN, USA). Serum SCC and ProGRP levels were analyzed by the ARCHITECT automated assay (Abbott Laboratories, Chicago, IL, USA). NSE was measured by a commercial electrochemiluminescence analyzer (Roche Diagnostics, Mannheim, Germany).

### Statistical analysis

In this study, all statistical analyses were obtained by SPSS 26 software. The diagnostic values of biomarkers were evaluated by the receiver operating characteristic (ROC) curve, and the ROC curve was drawn by Medcalc 16.8.4 software. Chemotactic cut-offs were calculated using the Youden index. Independent-sample T-test was used to analyze the relationship between serum biomarkers levels and pathological clinic features. T-test was also used to analyze the differences in serum biomarkers of different groups. The data were considered statistically significant when *P*-values were less than 0.05.

## Results

### Subject characteristics

A total of 294 patients and clinical features in this work were detailed in **Table 1.** There were 116 patients with benign lung diseases and 178 patients with lung cancer, 55 smokers in the benign lung disease group and 89 in the lung cancer group. The number of cases found in stages I and II and III and IV of TNM was 7, 14, 30, and 127, respectively. Among the patients with lung cancer, there were 40 cases of LADC, 64 of SQCLC, 33 of SCLC, and 41 patients with unknown pathological type. There was no significant difference in age and sex between benign lung disease and lung cancer groups.

### Serum levels of biomarkers in patients with lung cancer and benign lung diseases

**Figure 2:**
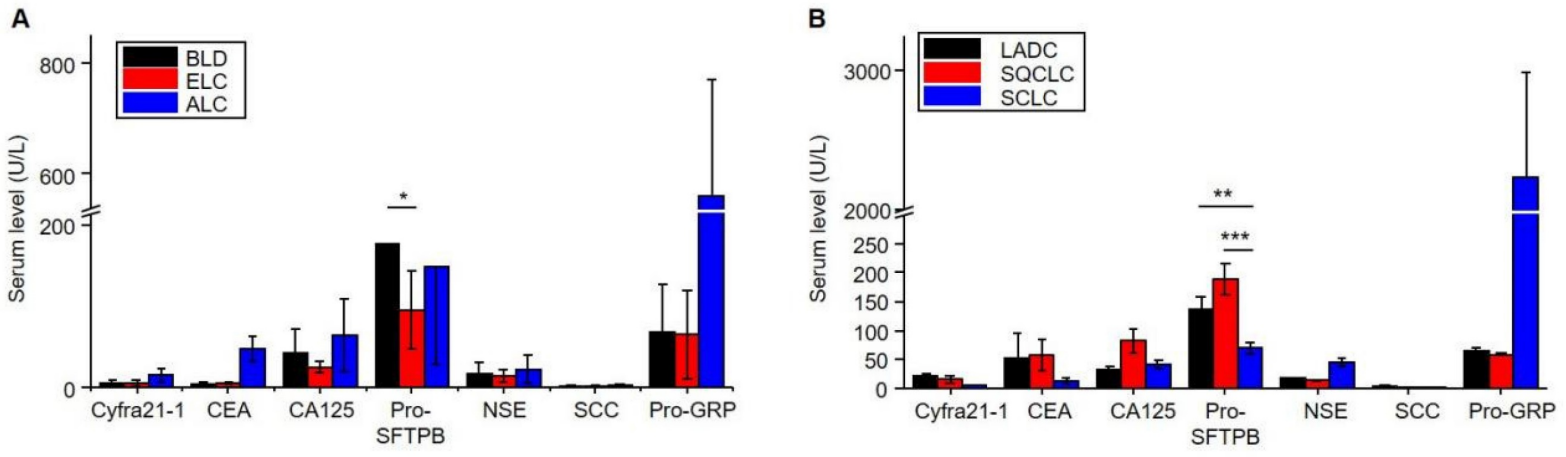
The serum levels of seven markers in patients with benign lung disease and lung cancer. A. Distribution in benign lung disease (BLD), early lung cancer (ELC), and advanced lung cancer (ALC). B. Distribution in lung adenocarcinoma (LADC), squamous-cell carcinoma (SQCLC), and small-cell lung cancer (SCLC). The data show mean value and standard deviations (SD). *P<0.05, **P<0.01 and ***P<0.001.

This study evaluated the serum protein levels of seven markers between the different groups. As shown in **Figure 2A** and **Table S1,** there were significant differences in serum Cyfra21-1 (*P* = 0.003), CEA (*P* = 0.008), NSE (*P* = 0.036), and Pro-GRP (*P* = 0.006) levels among the benign lung disease and lung cancer. Further, there were significant differences in serum Pro-SFTPB (*P* = 0.007) levels among benign lung disease and early lung cancer. This result indicated that Pro-SFTPB might be able to distinguish early lung cancer from benign lung disease.

To further explore the relationship between serum levels of protein markers and different pathological types of lung cancer, the present study evaluated the serum levels of seven proteins in LADC, SQCLC, and SCLC. **Table S2** shows significant differences in serum CA125 (*P*=0.026) and SCC (*P* = 0.018) levels among the LADC and SQCLC. Further, there were significant differences in serum Pro-SFTPB (*P* = 0.000), NSE (*P* = 0.000), SCC (*P* = 0.014), and Pro-GRP (*P* = 0.008) levels among the SQCLC and SCLC. The significant differences were shown in serum Cyfra21-1 (*P* = 0.003), Pro-SFTPB (*P* = 0.003), NSE (*P* = 0.001), SCC (*P* = 0.004), and Pro-GRP (*P* = 0.008) levels among the LADC and SCLC. As shown in **Figure 2B,** the serum Pro-SFTPB level of patients with non-small cell lung cancer was significantly higher than that of patients with small cell lung cancer. In comparison, the serum Pro-GRP level in the SCLC group was considerably higher than in the non-small cell lung cancer group. These results indicated that the multi-protein panel could distinguish LADC, SQCLC, and SCLC. In addition, the serum levels of the seven proteins were not significantly correlated with the patient's age, gender, smoking habit, and nodule size, as shown in **Table S3-S6**.

### Diagnosis performance of biomarkers panel distinguishing lung cancer and benign lung diseases

The diagnostic effects of combined and single detection of serum Cyfra21-1, CEA, CA125, and Pro-SFTPB levels (4MP) in benign lung disease and lung cancer patients were analyzed. In addition, the auxiliary diagnostic effect of serum NSE, SCC, and Pro-GRP levels (7MP) on the 4MP was evaluated.

**Figure 3:**
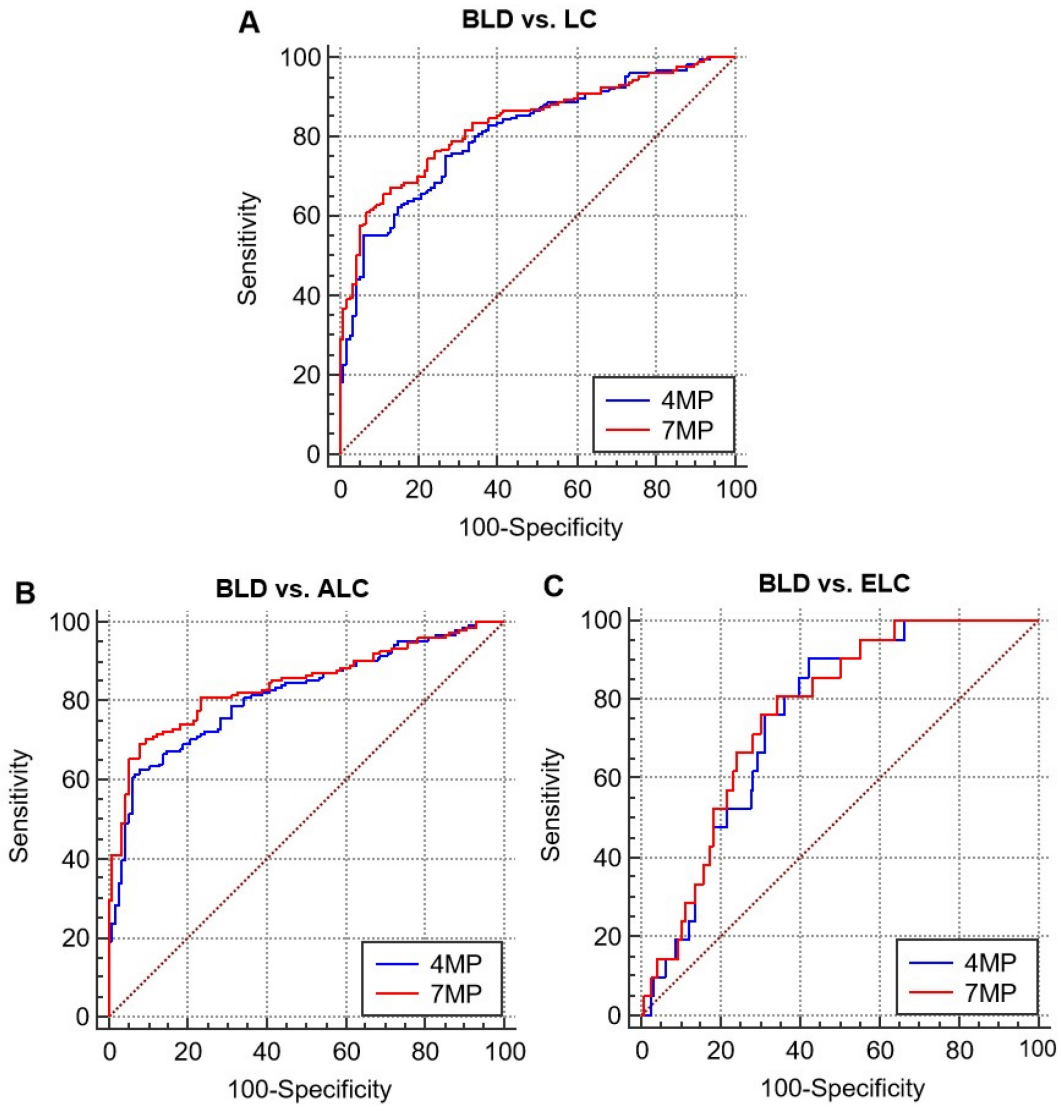
Receiver operating characteristic curves (ROCs) of 4MP and 7MP for differentiating patients with benign lung disease (BLD), lung cancer, early lung cancer (ELC), and advanced lung cancer (ALC).

As shown in **Figure 3 and Table 2**, the diagnostic effects of 4MP and 7MP were all higher than single detection of serum biomarkers levels. The area under the receiver operator characteristic curves (AUCs) of 4MP and 7MP in distinguishing benign lung disease and lung cancer were 0.808 (95% CI: 0.758-0.856) and 0.832 (95% CI: 0.786-0.878), respectively. But the AUCs of the single detection of biomarkers were only no more than 0.6 excluded CEA (AUC = 0.72). The AUCs of 4MP and 7MP in distinguishing benign lung disease and early lung cancer were 0.752 (95% CI: 0.662-0.843) and 0.764 (95% CI: 0.673-0.855), respectively. And the sensitivity of 4MP and 7MP was 90.48% and 81.00%, respectively. The AUCs of the single detection of biomarkers were only no more than 0.65. In distinguishing benign lung disease and advanced lung cancer, the AUCs of 4MP and 7MP were 0.816 (95% CI: 0.766-0.866) and 0.842 (95% CI: 0.795-0.889), respectively. And the specificity of 4MP and 7MP were all 92.24%. Thus, it can be concluded that the detection of serum NSE, SCC, and Pro-GRP levels was not apparent for the auxiliary diagnosis of 4MP in distinguishing benign lung disease and lung cancer. The diagnostic effect of the 4MP was already excellent enough. These results indicated that 4MP had the potential to be an auxiliary diagnosis biomarker panel in the early diagnosis of lung cancer.

### Diagnosis performance of biomarkers panel in distinguishing different pathological types of lung cancer

The diagnostic effects of combined and single detection of 4MP in different pathological types of lung cancer patients were analyzed. In addition, the auxiliary diagnostic effect of 7MP on the 4MP was evaluated.

**Figure 4:**
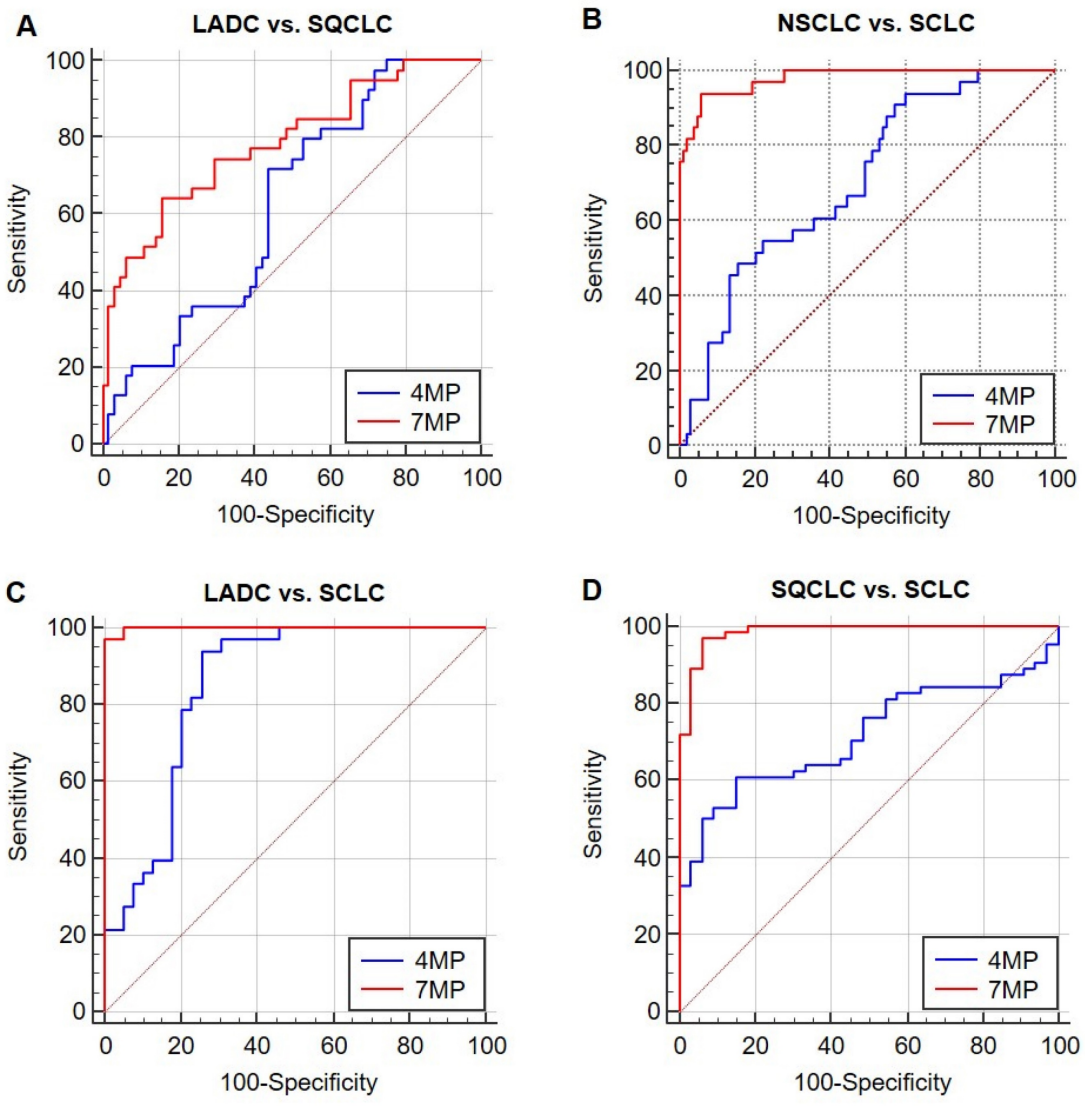
Receiver operating characteristic curves (ROCs) of 4MP and 7MP for differentiating patients with lung adenocarcinoma (LADC), squamous-cell carcinoma (SQCLC) and small cell lung cancer (SCLC).

As shown in **Figure 4** and **Table 3**, in distinguishing two types of NSCLC, the AUCs of 4MP and 7MP were 0.627 (95% CI: 0.519-0.734) and 0.784 (95% CI: 0.691-0.877), respectively. The sensitivity of 4MP and 7MP was 72.50% and 64.10%, respectively. The specificity of 4MP and 7MP was 56.25% and 84.37%, respectively. In this cohort, 7MP could serve as a supplementary detection panel for 4MP. In distinguishing SQCLC and SCLC, the AUCs of 4MP and 7MP were 0.716 (95% CI: 0.617-0.816) and 0.985 (95% CI: 0.966-1.005), respectively. The sensitivity of 4MP and 7MP was 60.94% and 96.87%, respectively. The specificity of 4MP and 7MP was 84.85% and 93.94%, respectively. In distinguishing LADC and SCLC, the AUCs of 4MP and 7MP were 0.849 (95% CI: 0.758-0.940) and 0.998 (95% CI: 0.994-1.003), respectively. The sensitivity of 4MP and 7MP was 93.94% and 96.97%, respectively. The specificity of 4MP and 7MP was 72.50% and 100.00%, respectively. In distinguishing NSCLC and SCLC, the AUCs of 4MP and 7MP were 0.705 (95% CI: 0.609-0.801) and 0.979 (95% CI: 0.956-1.001), respectively. The sensitivity and specificity of 4MP was 93.94% and 39.42%, respectively. The sensitivity and specificity of 7MP was 93.94% and 94.17%, respectively. These results show that the diagnosis performance of 7MP is significantly better than 4MP in distinguishing NSCLC and SCLC.

## Discussion

Many patients with lung cancer have metastatic symptoms. Early diagnosis is a prerequisite for improving the survival and prognosis of patients. Compared with chest X-rays, using LDCT for lung cancer screening demonstrated the benefits of reducing mortality. However, due to the limitations of imaging technology, the false positive rate of the results is high 15. In recent years, as an ideal minimally invasive and easy-to-collect medium, blood samples have been viral in cancer diagnosis.

CEA and NSE are commonly used clinical lung cancer protein markers, but the early diagnosis results are unsatisfactory 13. In addition, many studies have shown that lung cancer has many potential protein markers, but there are few candidates with both early cancer specificity and detection sensitivity 16. Many protein markers perform excellently in distinguishing healthy controls from patients with lung cancer, but there are not enough candidates for clinical translation. The clinical situation shows that the symptoms of many patients with lung cancer are similar to those of benign lung diseases such as pneumonia and pulmonary nodules, easy to cause misdiagnosis and miss the best treatment time 10. However, the current clinical and ongoing markers cannot accurately distinguish between lung cancer and benign lung diseases. Our collaborators previously studied the joint detection of a panel of four markers of CEA, CA125, Cyfra21-1, and Pro-SFTPB 17. The results showed that the 4MP distinguished lung cancer especially smoking patients from healthy controls (AUC 0.83, specificity 0.83, sensitivity 0.42). In addition, Pro-SFTPB showed excellent AUC diagnostic performance in identifying different subtypes of lung cancer. In a recent study, combinations of the four serum biomarkers have effectively predicted early lung cancer risk in patients with a smoking history (AUC 0.78, specificity 0.44, sensitivity 0.99) 18. Another study evaluated the performance of this panel in differentiating benign and malignant pulmonary nodules. The results showed that the combination of 4MP and nodule size had higher AUC than the model based on nodule size alone (4MP+nodule size, AUC 0.86; nodule size, AUC 0.897) 19. Our study well complements the clinical application of 4MP in the detection of benign and malignant lesions.

Many studies are mainly based on 4MP in the diagnosis of benign and malignant pulmonary nodules, and there are few studies on the differential diagnosis of overall benign lung diseases and lung cancer. This study aimed to explore the diagnostic performance of the 4MP in distinguishing benign pulmonary diseases from lung cancer, as well as in different lung cancer pathological types. This study also discussed the auxiliary role of conventional lung cancer diagnostic markers CEA, NSE, and Pro-GRP for the 4MP in early diagnosis of lung cancer. The results of this study show that the 4MP has an excellent diagnostic performance. Especially in distinguishing early lung cancer and benign lung diseases, the detection sensitivity is the highest, up to 90.48%. And the diagnostic performance of the 4MP is better than that of the single marker, and the performance of the 7MP is not much better than 4MP. According to the performance of individual markers, the contribution of Pro-SFTPB is mainly in identifying early lung cancer and benign lung diseases.

The most significant contribution to the identification of advanced lung cancer and benign lung diseases is Pro-GRP, which is abnormally elevated in serum levels in advanced cancer. It has been previously reported that there is a significant and independent correlation between plasma Pro-SFTPB and lung cancer, and it also plays a predictive role in lung cancer 20. Pro-SFTPB is associated with early lung cancer and is elevated in the blood circulation of people at high risk of lung cancer, but the exact mechanism is not clear. Some studies have shown that SFTPB is initially synthesized by alveolar lung cells and non-ciliated bronchioles 20, 21. During synthesis, Pro-SFTPB is hydrolyzed and cleaved by protein in the endoplasmic reticulum, resulting in the synthesis and secretion of mature SFTPB 22. However, the imbalance of SFTPB synthesis in lung cancer cells leads to the over-expression of Pro-SFTPB.

To further explore the stratification ability of the 4MP on different pathological types, the differential performance of the 4MP and 7MP was compared. The experimental results show that combining three conventional lung cancer markers with 4MP can significantly improve diagnostic performance. It means that the NSE, SCC, and Pro-GRP can be used as resultful auxiliary detection items of the 4MP, which can effectively help the 4MP to complete the stratification of different lung cancer subtypes. Pro-GRP, a peptide secreted by tumor cells, is an effective marker for the progression of SCLC 23. NSE is a tumor biomarker found in patients with SCLC 24. A past study showed the AUC of Pro-GRP and NSE to distinct SCLC and NSCLC was 0.93 and 0.79, respectively 25. Our results showed that the 7MP was less practical than SCLC and NSCLC in identifying LADC and SQCLC. The AUC was 0.784 in identifying LADC and SQCLC, while the AUC was 0.985 and 0.998, respectively, in distinguishing LADC and SQCLC from SCLC. In differentiating NSCLC from SCLC, the AUC was 0.979, and the sensitivity and specificity were up to 93.94% and 94.17, respectively. This suggests that we can carry out 7MP for patients diagnosed with lung cancer as an auxiliary diagnosis of pathological type identification to help achieve an accurate diagnosis and personalized treatment for patients with lung cancer.

This study also has some limitations. The sample size of early-stage lung cancer and SCLC is still insufficient. In addition, there were insufficient samples for other types of NSCLC. Furthermore, the stratification of benign lung disease was not done deeply enough. In the future, this study will conduct a stratified analysis of lung cancer and specific benign diseases in a more complete large-scale cohort to further explore the performance of 4MP in the auxiliary diagnosis of lung cancer. Further exploration of more early lung cancer specific markers to assist 4MP to improve the detection specificity.

## Conclusion

In summary, the multiple biomarker combination of Pro-SFTPB, CA125, Cyfra21-1, and CEA can distinguish lung cancer from benign lung disease, which performs better than a single biomarker. Traditional lung cancer biomarkers NSE, SCC, and Pro-GRP can significantly improve the performance of 4MP in the differentiation of LADC, SQCLC, and SCLC, which is expected to contribute to the accurate diagnosis and personalized treatment of patients with lung cancer. It is hoped that the results the results of this study lay the foundation for large-scale clinical trials before clinical transformation, and further provide clinical feasibility for early diagnosis of lung cancer in China.

## Supplementary Material

Supplementary tables.

## Figures and Tables

**Table 1:** The baseline chart of patients in this study.

		All (n=294)		BLD (n=116)		LC (n=178)		*P*
		n	%	n	%	n	%	
Gender	male	181	61.56%	71	61.21%	110	61.80%	0.919
	female	113	38.44%	45	38.79%	68	38.20%	
Age	>= 45	280	95.24%	107	92.24%	173	97.19%	0.043
	<45	14	4.76%	9	7.76%	5	2.81%	
Smoke stage	yes	144	48.98%	55	47.41%	89	50.00%	0.665
	no	150	51.02%	61	52.59%	89	50.00%	
Nodules size (mm)	>= 8					67	37.64%	
	<8					12	6.74%	
	unknown					99	55.62%	
Stage	I					7	3.93%	
	II					14	7.87%	
	III					30	16.85%	
	IV					127	71.35%	
Classification	LADC					40	22.47%	
	SQCLC					64	35.96%	
	SCLC					33	18.54%	
	unknown					41	23.03%	

**Table 2:** The discrimination performance of 4MP and 7MP for patients with benign lung disease (BLD), lung cancer, early lung cancer (ELC) and advanced lung cancer (ALC).

Group	Biomarkers	AUC	*P*	95% CI		Sensitivity	Specificity
				Low	High		
BLD vs. LC	Cyfra21-1	0.677	0.000	0.616	0.737		
	CEA	0.720	0.000	0.663	0.777		
	CA125	0.550	0.145	0.483	0.618		
	Pro-SFTPB	0.554	0.126	0.491	0.608		
	NSE	0.521	0.538	0.455	0.587		
	SCC	0.583	0.015	0.516	0.651		
	Pro-GRP	0.554	0.107	0.488	0.619		
	**4MP**	**0.808**	**0.000**	**0.758**	**0.856**	**55.62%**	**93.97%**
	**7MP**	**0.832**	**0.000**	**0.786**	**0.878**	**65.54%**	**88.79%**
BLD vs. ELC	Cyfra21-1	0.574	0.243	0.45	0.699		
	CEA	0.545	0.461	0.425	0.665		
	CA125	0.652	0.018	0.566	0.731		
	Pro-SFTPB	0.645	0.015	0.559	0.725		
	NSE	0.612	0.124	0.469	0.755		
	SCC	0.513	0.823	0.398	0.628		
	Pro-GRP	0.504	0.954	0.372	0.635		
	**4MP**	**0.752**	**0.000**	**0.662**	**0.843**	**90.48%**	**57.76%**
	**7MP**	**0.764**	**0.000**	**0.673**	**0.855**	**81.00%**	**61.50%**
BLD vs. ALC	Cyfra21-1	0.690	0.000	0.629	0.752		
	CEA	0.744	0.000	0.686	0.801		
	CA125	0.577	0.028	0.508	0.647		
	Pro-SFTPB	0.538	0.250	0.476	0.598		
	NSE	0.508	0.808	0.440	0.577		
	SCC	0.593	0.008	0.524	0.661		
	Pro-GRP	0.560	0.080	0.493	0.628		
	**4MP**	**0.816**	**0.000**	**0.766**	**0.866**	**63.06%**	**92.24%**
	**7MP**	**0.842**	**0.000**	**0.795**	**0.889**	**69.23%**	**92.24%**

**Table 3:** The discrimination performance of 4MP and 7MP for patients with lung adenocarcinoma (LADC), squamous-cell carcinoma (SQCLC) and small cell lung cancer (SCLC).

Group	Biomarkers	AUC	*P*	95% CI		Sensitivity	Specificity
				Low	High		
LADC vs. SQCLC	Cyfra21-1	0.664	0.004	0.551	0.777		
	CEA	0.688	0.000	0.584	0.792		
	CA125	0.565	0.251	0.454	0.677		
	Pro-SFTPB	0.513	0.813	0.402	0.625		
	NSE	0.609	0.054	0.498	0.721		
	SCC	0.742	0.000	0.641	0.842		
	Pro-GRP	0.566	0.265	0.450	0.681		
	**4MP**	**0.627**	**0.021**	**0.519**	**0.734**	**72.50%**	**56.25%**
	**7MP**	**0.784**	**0.000**	**0.691**	**0.877**	**64.10%**	**84.37%**
SQCLC vs. SCLC	Cyfra21-1	0.527	0.646	0.412	0.642		
	CEA	0.667	0.003	0.556	0.778		
	CA125	0.531	0.606	0.414	0.648		
	Pro-SFTPB	0.697	0.000	0.593	0.801		
	NSE	0.775	0.000	0.669	0.881		
	SCC	0.616	0.047	0.501	0.732		
	Pro-GRP	0.911	0.000	0.838	0.984		
	**4MP**	**0.716**	**0.000**	**0.617**	**0.816**	**60.94%**	**84.85%**
	**7MP**	**0.985**	**0.000**	**0.966**	**1.005**	**96.87%**	**93.94%**
LADC vs. SCLC	Cyfra21-1	0.761	0.000	0.644	0.879		
	CEA	0.527	0.694	0.392	0.663		
	CA125	0.558	0.401	0.423	0.693		
	Pro-SFTPB	0.717	0.000	0.598	0.836		
	NSE	0.707	0.001	0.581	0.833		
	SCC	0.833	0.000	0.739	0.926		
	Pro-GRP	0.895	0.000	0.812	0.978		
	**4MP**	**0.849**	**0.000**	**0.758**	**0.940**	**93.94%**	**72.50%**
	**7MP**	**0.998**	**0.000**	**0.994**	**1.003**	**96.97%**	**100.00%**
NSCLC vs. SCLC	Cyfra21-1	0.616	0.015	0.522	0.709		
	CEA	0.594	0.086	0.487	0.700		
	CA125	0.503	0.960	0.394	0.612		
	Pro-SFTPB	0.705	0.000	0.609	0.801		
	NSE	0.749	0.000	0.641	0.858		
	SCC	0.698	0.000	0.605	0.792		
	Pro-GRP	0.905	0.000	0.830	0.980		
	**4MP**	**0.705**	**0.000**	**0.609**	**0.801**	**93.940**	**39.420**
	**7MP**	**0.979**	**0.000**	**0.956**	**1.001**	**93.940**	**94.170**

## Abbreviations

BLD: benign lung disease
LADC: lung adenocarcinoma
SQCLC: squamous-cell carcinoma
SCLC: small-cell lung cancer
Pro-SFTPB: pro-surfactant protein B
CA125: carbohydrate antigen 125
Cyfra21-1: cytokeratin 19 fragment
CEA: carcinoembryonic antigen
NSE: neuron specific enolase
SCC: squamous-cell carcinoma antigen
Pro-GRP: pro-gastrin-releasing peptide
ROC: receiver operator characteristic curve
AUC: area under the ROC
