# Supplementary material for: Multi-Biomarkers Panel in Identifying Benign and Malignant Lung Diseases and Pathological Types of Lung Cancer
Source: J Cancer. 2023 Jun 26;14(10):1904–12. doi: 10.7150/jca.85846 (PMC10355209; doi:10.7150/jca.85846)
Supplement: Supplementary file 1 — Supplementary tables. [file jcav14p1904s1.pdf]

Table S1. The serum levels of seven markers in patients with benign lung disease (BLD), early lung cancer (ELC) and advanced lung cancer (ALC).

Biomarkers-	Group	N	Mean	SD	*P*
Cyfra21-1	BLD	116	5.31	0.93	0.003
	LC	178	13.11	2.45	
CEA	BLD	116	3.56	0.40	0.008
	LC	178	42.25	14.37	
CA125	BLD	116	42.54	6.47	0.168
	LC	178	59.07	8.68	
Pro-SFTPB	BLD	116	176.53	17.65	0.106
	LC	178	141.94	12.80	
NSE	BLD	116	16.03	1.04	0.036
	LC	178	20.67	1.94	
SCC	BLD	116	1.49	0.22	0.054
	LC	177	2.26	0.34	
Pro-GRP	BLD	116	68.02	4.86	0.006
	LC	177	500.45	155.49	
Cyfra21-1	BLD	116	5.31	0.93	0.895
	ELC	21	5.01	0.79	
CEA	BLD	116	3.56	0.40	0.310
	ELC	21	4.76	1.66	
CA125	BLD	116	42.54	6.47	0.260
	ELC	21	24.80	8.67	
Pro-SFTPB	BLD	116	176.53	17.65	0.007
	ELC	21	94.91	22.79	
NSE	BLD	116	16.03	1.04	0.385
	ELC	21	13.69	2.64	
SCC	BLD	116	1.49	0.22	0.597
	ELC	21	1.21	0.25	
Pro-GRP	BLD	116	68.02	4.86	0.740
	ELC	21	64.15	4.85	
Cyfra21-1	ELC	21	5.01	0.79	0.228
	ALC	157	14.19	2.77	
CEA	ELC	21	4.76	1.66	0.341
	ALC	157	47.27	16.25	
CA125	ELC	21	24.80	8.67	0.150
	ALC	157	63.65	9.73	
Pro-SFTPB	ELC	21	94.91	22.79	0.180
	ALC	157	148.23	14.13	
NSE	ELC	21	13.69	2.64	0.190
	ALC	157	21.60	2.16	
SCC	ELC	21	1.21	0.25	0.253

	ALC	156	2.40	0.38	
Pro-GRP	ELC	21	64.15	4.85	0.305
	ALC	156	559.18	175.96	

Table S2. The serum levels of seven markers in patients with lung adenocarcinoma (LADC), squamous-cell carcinoma (SQCLC) and small-cell lung cancer (SCLC).

Biomarkers	Group	N	Mean	SD	*P*
Cyfra21-1	LADC	40	20.27	4.69	0.503
	SQCLC	64	14.67	5.88	
CEA	LADC	40	51.14	44.68	0.900
	SQCLC	64	57.31	27.04	
CA125	LADC	40	33.37	5.02	0.026
	SQCLC	64	82.09	20.82	
Pro-SFTPB	LADC	40	137.55	19.86	0.133
	SQCLC	64	188.43	27.12	
NSE	LADC	40	17.22	2.17	0.068
	SQCLC	64	13.01	1.20	
SCC	LADC	39	3.90	0.99	0.018
	SQCLC	64	1.42	0.19	
Pro-GRP	LADC	39	65.75	4.12	0.156
	SQCLC	64	58.85	2.81	
Cyfra21-1	SQCLC	64	14.67	5.88	0.255
	SCLC	33	5.27	0.44	
CEA	SQCLC	64	57.31	27.04	0.234
	SCLC	33	11.95	4.49	
CA125	SQCLC	64	82.09	20.82	0.067
	SCLC	33	40.98	7.38	
Pro-SFTPB	SQCLC	64	188.43	27.12	0.000
	SCLC	33	69.85	8.93	
NSE	SQCLC	64	13.01	1.20	0.000
	SCLC	33	44.42	7.19	
SCC	SQCLC	64	1.42	0.19	0.014
	SCLC	33	0.91	0.06	
Pro-GRP	SQCLC	64	58.85	2.81	0.008
	SCLC	33	2227.44	764.49	
Cyfra21-1	LADC	40	20.27	4.69	0.003
	SCLC	33	5.27	0.44	
CEA	LADC	40	51.14	44.68	0.430
	SCLC	33	11.95	4.49	
CA125	LADC	40	33.37	5.02	0.384
	SCLC	33	40.98	7.38	
Pro-SFTPB	LADC	40	137.55	19.86	0.003
	SCLC	33	69.85	8.93	
NSE	LADC	40	17.22	2.17	0.001

	SCLC	33	44.42	7.19	
SCC	LADC	39	3.90	0.99	0.004
	SCLC	33	0.91	0.06	
Pro-GRP	LADC	39	65.75	4.12	0.008
	SCLC	33	2227.44	764.49	

Table S3. The correlation analysis between the serum levels of seven markers and smoking habit.

Biomarkers	Smoking habit					
	Smoking habit	N	Mean	*P*	95% CI	
					Low	High
Cyfra21-1	no	89	11.68	0.759	-27.714	37.919
	yes	89	14.53			
CEA	no	89	49.05	0.529	-253.593	130.759
	yes	89	35.45			
CA125	no	89	48.94	0.476	-158.061	74.061
	yes	89	69.20			
Pro-SFTPB	no	89	123.44	0.526	-970.105	613.627
	yes	89	160.44			
NSE	no	89	19.86	0.829	-23.161	28.849
	yes	89	21.47			
SCC	no	89	1.51	0.543	-3.098	5.868
	yes	88	3.02			
Pro-GRP	no	89	377.70	0.676	-1635.331	2515.890
	yes	88	624.59			

Table S4. The correlation analysis between the serum levels of seven markers and gender.

Biomarkers	Gender					
	Gender	N	Mean	*P*	95% CI	
					Low	High
Cyfra21-1	male	110	14.18	0.579	-7.167	12.787
	female	68	11.37			
CEA	male	110	49.17	0.542	-40.351	76.569
	female	68	31.06			
CA125	male	110	69.01	0.086	-3.719	55.792
	female	68	42.98			
Pro-SFTPB	male	110	149.87	0.432	-31.281	72.807
	female	68	129.11			
NSE	male	110	21.51	0.584	-5.707	10.102
	female	68	19.31			
SCC	male	109	3.01	0.000	0.880	3.020
	female	68	1.06			
Pro-GRP	male	109	553.66	0.666	-493.871	770.914
	female	68	415.14	0.579	-7.167	12.787

Table S5. The correlation analysis between the serum levels of seven markers and age.

Biomarkers	Age					
	Age	N	Mean	*P*	95% CI	
					Low	High
Cyfra21-1	>= 45	173	13.27	0.562	-12.549	6.840
	< 45	4	8.17			
CEA	>= 45	173	41.09	0.637	-43.228	70.439
	< 45	4	102.51			
CA125	>= 45	173	58.43	0.245	-54.496	13.987
	< 45	4	100.43			
Pro-SFTPB	>= 45	173	138.53	0.149	-87.366	13.361
	< 45	4	316.77			
NSE	>= 45	173	20.77	0.679	-9.298	6.071
	< 45	4	17.92			
SCC	>= 45	172	2.30	0.026	-2.825	-0.187
	< 45	4	0.92			
Pro-GRP	>= 45	172	513.01	0.429	-861.318	367.537
	< 45	4	72.73			

Table S6. The correlation analysis between the serum levels of seven markers and nodule size.

Biomarkers	Nodule size					
	Nodule size	N	Mean	*P*	95% CI	
					Low	High
Cyfra21-1	>= 8	67	11.85	0.613	-10.295	17.335
	< 8	12	8.33			
CEA	>= 8	67	66.15	0.540	-119.175	225.849
	< 8	12	12.81			
CA125	>= 8	67	51.05	0.347	-31.428	88.377
	< 8	12	22.58			
Pro-SFTPB	>= 8	67	124.77	0.930	-80.869	88.382
	< 8	12	121.01			
NSE	>= 8	67	15.94	0.344	-40.037	15.118
	< 8	12	28.40			
SCC	>= 8	67	2.98	0.283	-1.701	5.736
	< 8	12	0.96			
Pro-GRP	>= 8	67	219.37	0.253	-1244.358	332.427
	< 8	12	675.34			
